# Personality as an intermediate phenotype for genetic dissection of alcohol use disorder

**DOI:** 10.1007/s00702-016-1672-9

**Published:** 2017-01-04

**Authors:** Lars Oreland, Gianvito Lagravinese, Simone Toffoletto, Kent W. Nilsson, Jaanus Harro, C. Robert Cloninger, Erika Comasco

**Affiliations:** 1 Department of Neuroscience, Uppsala University, BMC, Box 593, 751 24 Uppsala, Sweden; 2 Centre for Clinical Research, Uppsala University, Västmanland County Counci, Västerås, Sweden; 3 Division of Neuropsychopharmacology, Department of Psychology, University of Tartu, Tartu, Estonia; 4 Psychiatry Clinic, North Estonia Medical Centre, Tallinn, Estonia; 5 Department of Psychiatry, School of Medicine, Washington University, St. Louis, MO USA

**Keywords:** Alcohol, AUD, Gene, Personality, Serotonin

## Abstract

Genetic and environmental interactive influences on predisposition to develop alcohol use disorder (AUD) account for the high heterogeneity among AUD patients and make research on the risk and resiliency factors complicated. Several attempts have been made to identify the genetic basis of AUD; however, only few genetic polymorphisms have consistently been associated with AUD. Intermediate phenotypes are expected to be in-between proxies of basic neuronal biological processes and nosological symptoms of AUD. Personality is likely to be a top candidate intermediate phenotype for the dissection of the genetic underpinnings of different subtypes of AUD. To date, 38 studies have investigated personality traits, commonly assessed by the Cloninger’s Tridimensional Personality Questionnaire (TPQ) or Temperament and Character Inventory (TCI), in relation to polymorphisms of candidate genes of neurotransmitter systems in alcohol-dependent patients. Particular attention has been given to the functional polymorphism of the serotonin transporter gene (5-HTTLPR), however, leading to contradictory results, whereas results with polymorphisms in other candidate monoaminergic genes (e.g., tryptophan hydroxylase, serotonin receptors, monoamine oxidases, dopamine receptors and transporter) are sparse. Only one genome-wide association study has been performed so far and identified the *ABLIM1* gene of relevance for novelty seeking, harm avoidance and reward dependence in alcohol-dependent patients. Studies investigating genetic factors together with personality could help to define more homogenous subgroups of AUD patients and facilitate treatment strategies. This review also urges the scientific community to combine genetic data with psychobiological and environmental data to further dissect the link between personality and AUD.

## Introduction

Alcohol use disorder (AUD), a psychiatric disorder characterized by excessive and uncontrolled drinking that causes harm and distress, has devastating consequences for men and women of all ages. According to recent statistics, AUD is among the four most disabling diseases, affecting about 14.6 million persons in Europe (Wittchen et al. 2011). AUD is a heterogeneous disorder, which results from the interplay between both genetic and environmental factors (Goldman et al. 2005). To date, there are only four drugs approved by the Food and Drug Administration and four off-label drugs commonly used for AUD, mainly because the putative target(s) and biological underpinnings of AUD are only fragmentarily known (Franck and Jayaram-Lindström 2013; Baingana et al. 2015).

**Fig. 1:**
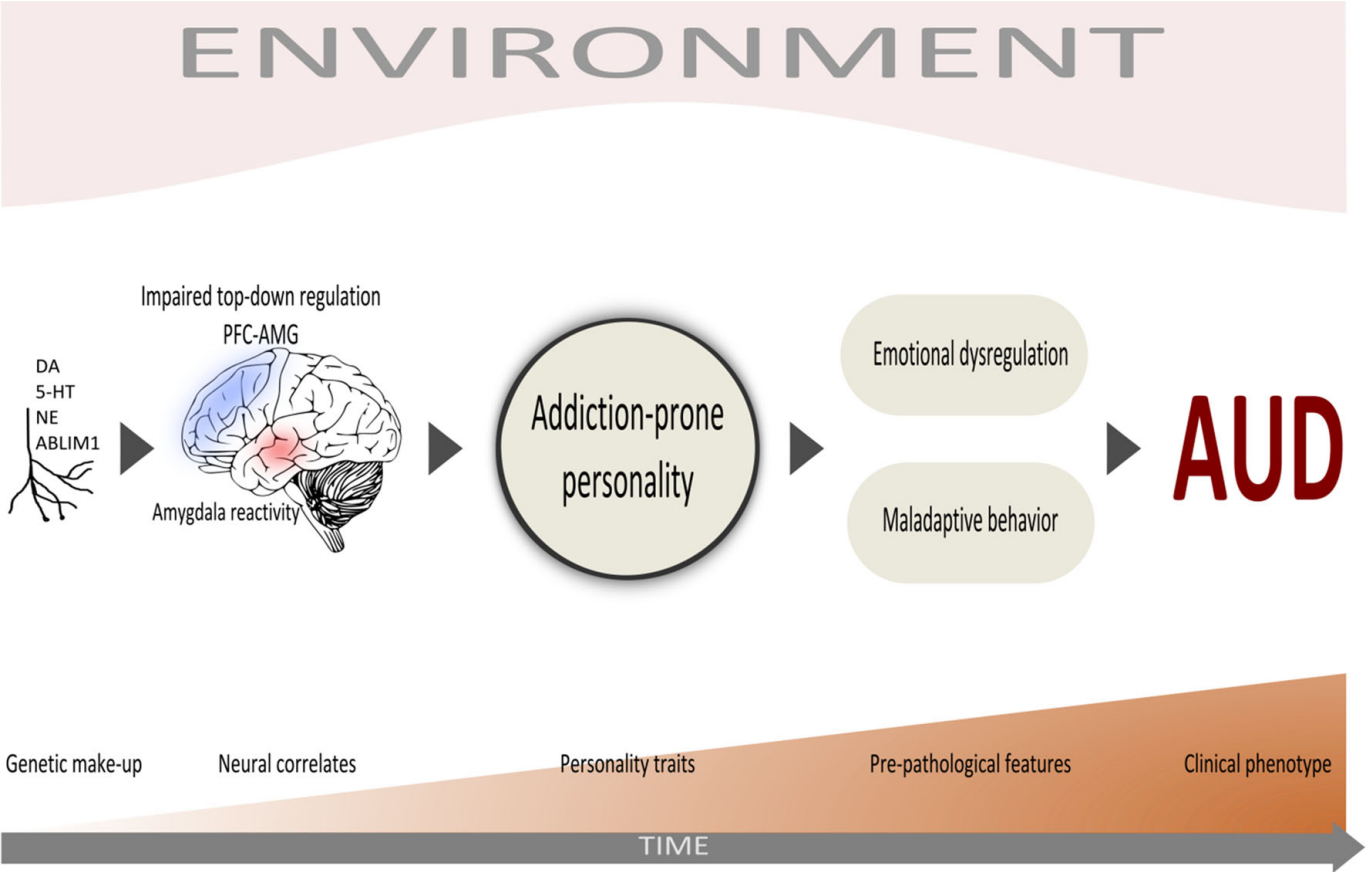
The proposed study model of personality traits as intermediate phenotype involved in the development of AUD. *ABLIM1* Actin Binding LIM Protein 1, *AMG* amygdala, *AUD* alcohol use disorder, *DA* dopamine, *NE* noradrenaline, *PFC* prefrontal cortex, *5-HT* serotonin

Some aspects related to AUD, such as personality, cognitive function, alcohol metabolism and underlying neurophysiology, are partially regulated by genetic factors that likely influence disease susceptibility (Hines et al. 2005). The genetic architecture of intermediate phenotypes, such as personality traits, may be easier to dissect than clinical end points (Rasetti and Weinberger 2011; Meyer-Lindenberg and Weinberger 2006; Almasy 2003). Genetic factors indeed seem to account for one-third to one-half of the inter-individual differences in personality (Slutske et al. 2002) and about 50% of the heritability of AUD (Goldman et al. 2005). Nevertheless, as AUD is clinically defined, genetic predictors as such may be weaker than clinical ones with regard to prognosis or treatment response. Thus, the investigation of genetic factors together with personality could help define more homogenous subtypes of AUD leading to potential operational diagnostic categories (Fig. 1).

Through the years, many models to classify clinically relevant subtypes have been proposed to improve the clinical management of the patients (Leggio et al. 2009). Particularly, three typologies of alcoholism, e.g., Cloninger’s typology, Lesch alcohol typology (Lesch and Walter 1996) and NETER alcoholism typology (Cardoso et al. 2006) have been largely studied in relation to genetic influence, biological markers, personality, clinical features and prognosis (Pombo et al. 2015). Mainly three dimensions of personality have been found to be involved in individuals with AUD, namely impulsivity/novelty seeking, neuroticism/negative emotionality and extraversion/reward dependence [for review; see Mulder (2002)].

Among these, the psychobiological Cloninger’s model, built on Swedish adoption studies of children of alcoholics, has been widely studied and validated (Pombo et al. 2015), as in the herein reviewed studies. It defines two alcoholism subtypes, type I and type II, which could be distinguished as having distinct genetic and environmental causes (Cloninger et al. 1981). Type I is characterized by late onset, a low degree of heritability, few social complications and strong environmental influence. Individuals with this type of alcoholism tend to drink alcohol as self-medication. Type II is characterized by early onset and strong genetic influence, comorbid substance use and abuse, and social complications such as a family history of alcoholism, antisocial behavior and depression (Cloninger et al. 1981). Of interest is that these two subtypes are characterized by different personality traits, as shown by Oreland, von Knorring and co-workers in 1985 (Knorring et al. 1985; von Knorring et al. 1991). Cloninger later developed the TCI scale and found that novelty seeking, harm avoidance and reward dependence were associated with dopamine (DA), serotonin (5-HT) and norepinephrine (NA) neurotransmitter systems, respectively. Typical for type I is low novelty seeking (NS), high harm avoidance (HA) and high reward dependence (RD), while individuals with type II have the reverse characteristics: high NS, low HA and low RD (Cloninger et al. 1996). The predicted role of these temperament dimensions has been confirmed in prospective longitudinal studies of childhood personality (Cloninger et al. 1988), family studies (Grucza et al. 2006) and national probability samples (Cloninger et al. 1995).

A different genetic background may most likely underlie this personality-based dichotomy of AUD. Hence, the purpose of this review is to examine genetic factors contributing to variations in dimensions of personality triggering the development of AUD. Pinpointing genes affecting variations in quantitative measures of intermediate phenotypes such as personality may facilitate the characterization of vulnerability factors.

## Materials and methods

A computerized search of the literature was performed to identify all studies of genetic polymorphism and personality in alcohol-dependent patients (ADP) published before August 2016. Articles have been retrieved from PubMed/MEDLINE and Google Scholar using the following search terms and relevant combinations of them: “Alcohol”, “alcoholism”, “genes”, “polymorphism”, “alcohol-dependent patients”, “Alcohol use disorder”,“AUD”, “Alcohol personality disorder”, “Alcohol intermediate phenotype”, “personality substance use disorder”, “SUD”, “personality traits”, “Type 1”, “Type 2”, “Novelty seeking”, “Harm avoidance”, “Serotonin transporter gene” and “5-HTTLPR”. The list of references cited in the retrieved articles was also used to identify relevant articles. Candidate gene, gene-by-gene interaction (G × G) and genome-wide association studies (GWAS), in which the association between genetic variants and standardized measures of personality among alcohol-dependent patients were examined and selected for the present review. For each study, the following information was extracted: first author, publication year, sample size, sex ratio, mean age, ethnicity, clinical diagnosis, personality measure, gene and polymorphism, allele/genotype frequencies by groups and the main findings regarding the association between polymorphism and personality. With regard to personality traits assessment, we included studies that have made use of standardized self-report questionnaires. As the polymorphisms in the serotonin transporter gene (*SLC6A4*) were the most investigated genetic variants, the studies were grouped into those that explored serotonin transporter-related polymorphisms (Table 1) and those that explored other genetic variants (Table 2). No statistical analyses were performed due to high heterogeneity between the studies.

**Table 1:** Serotonin transporter genotype, personality and AUD

Study	*N* (*M* %)	Age (mean ± SD)	Age of onset/duration	Ethnicity/nationality	Diagnosis	Personality scale/inventory	*SLC6A4* polymorphism	Allele/genotype frequencies by group	Association polymorphism–personality
Sander et al. (1998)	315 P (n.a.) 216 HC (n.a.)	n.a.	/	German	ICD-10 AD (315/315), AD\DPD (64/315)	TPQ (101 AD, including 39 AD\DPD)	5-HTTLPR	n.s.	↓ HA in AD\DPD with S/S vs S/L, L/L^§^ ↑ NS in AD\DPD^§^ with S/S, S/L vs L/L
Hallikainen et al. (1999)	114 P (100%)	43.8 ± 8.8	/	Finnish	DSM-III-R AD, CLON 1	/	5-HTTLPR	↑ S in CLON 2 vs CLON 1 and HC	S/S vs L/L >risk for CLON 2 vs HC [OR = 3.9] S/S vs L/S, L/L >risk for CLON 2 vs HC [OR = 3.14]
	51 P (100%)	30.1 ± 8.4	/	Finnish	DSM-III-R AD + DSM-IV ASPD, CLON 2	/			
	54 HC (100%)	44.1 ± 7.9	/	Finnish					
Matsushita et al. (2001)	692 P (100%) 270 HC (100%)	50.5 ± 9.7 50 ± 21.3	/	Japanese	DSM-III-R AD Group A (370/697): FEIGHNER clinical characteristics	Group B (322/697): TCI, SSS	5-HTTLPR	↑ S, S/S in AD with BD (group A)	n.s.^#^
Stoltenberg et al. (2002)	150 P (n.a.)	n.a.	/	Caucasian	FEIGHNER	NEO-FFI	5-HTTLPR	n.s.	↑ Openness—S/S, L/S vs L/L (only in males)
Wiesbeck et al. (2004)	124 P (65%)	41.3 ± 8.5	27.7 ± 9.5	Caucasian	ICD-10 AD	TCI	5-HTTLPR	n.s.	↑ HA—S/L vs L/L
Lin et al. (2007)	133 P (86%)	38.9 ± 8.9	4.7 ± 1.2	Han Chinese	DSM-IV ANX\DEP (87/133)	TPQ (NS, HA)	5-HTTLPR	n.s.	↑ NS—S/S in ANX\DEP vs PURE ↑ HA—S/L or L/L in ANX\DEP vs PURE
		40.7 ± 8.4	4.5 ± 1.2		DSM-IV PURE (46/133)				
	57 HC (n.a.)	n.a.							
Koller et al. (2008)	368 P (78%)	43.3 ± 10	31.2 ± 9	German	ICD 10 AD, DSM-IV AD	TCI	5-HTTLPR, STin2 17 bp VNTR	n.s.	↑ HA—S/12 (S + 12R haplotype) ↓ HA—L/9 (L + 9R haplotype)
Wu et al. (2008)	127 P inmates (100%)	30.7 ± 7.5	/	Han Chinese	DSM-IV AD\ASPD (43/127), ASPD (84/127)	TPQ (NS, HA)	5-HTTLPR	↑ S/S in AD\ASPD vs ASPD	↑ NS—S/S in AD\ASPD vs ASPD
Herman et al. (2011)	862 P (73%)	40 ± 11.1	26 ± 10.4	Caucasian (701), Afro-American (79), Hispanic (82)	DSM-III-R AD (9.2% also ASPD)	CPI-So (lower score = greater sociopathy)	rs25531	n.s.	↓ CPI-So—L_A_/L_A_ vs S/L_A_, L_G_/L_A_, S/S, S/L_G_, L_G_/L_G_ in males ↓ CPI-So—S/S, S/L_G_, L_G_/L_G_ vs L_A_/L_A_ in females
Wang et al. (2013)	102 P (89%)	39.1 ± 9	11.4 ± 8	Han Chinese	DSM-IV AD	TPQ (NS, HA)	rs25531, novel allelic variants (XL) [Low functional: SS, SL_G_, L_G_L_G;_ High functional: S/L_A_, L_G_/L_A_, L_A_/L_A_, S/XL, L_A_/XL, L_G_/XL]	↑ S/S, S/L_G_, L_G_/L_G_ in P vs HC	n.s. # (for stratified genotype analysis see Table 2)
	111 HC (81%)	36.9 ± 8.7							

*AD* alcohol dependence, *ALDH* aldehyde dehydrogenase, *ANX/DEP* anxiety-depressive alcohol dependence, *ASPD* antisocial personality disorder, *BD* binge drinking, *CLON 1* Cloninger Type I, *CLON 2* Cloninger Type II, *CPI-So* California Psychological Inventory, *DPD* dissocial personality disorder, *DRD2* dopamine receptor 2, *F* females, *HC* healthy controls, *ICD-10* international statistical classification of diseases and related health problems, *KSP* Karolinska Scales of Personality, *L* long allele, *M* males, *NEO-FFI* NEO-Five Factor Inventory, *NEO-PI* Neuroticism Extraversion Openness Personality Inventory, *P* patients, *PURE* pure alcohol dependence, *S* short allele, *SSS* Sensation Seeking Scale, *TCI* Temperament and Character Inventory, *TPQ* Tridimensional Personality Questionnaire, *HA* harm avoidance, *NS* novelty seeking, *RD* reward dependence, *VNTR* variable tandem number repeats, *n.a.* not available, *n.s.* non- significant

^§^ Non-significant after correction for multiple testing

^#^ Correction for multiple testing

^+^ Stratified genotype analysis

**Table 2:** Personality, alcoholism and polymorphic genetic markers

Study	*N* (*M* %)	Age (mean ± SD)	Ethnicity/nationality	Diagnosis	Personality scale/inventory	Gene	Polymorphism	Allele/genotype frequencies by groups	Association personality–polymorphism
Sander et al. (1997)	252 P (100%), 197 HC (n.a.)	41.9 ± 9.4 n.a.	German	ICD-10 AD (252/252), AD\DPD (56/252)	TPQ	*DRD4*	exon III 48 bp VNTR	n.s.	n.s.
Bau et al. (1999)	110 P (100%)	41	Caucasian	DSM-III AD	TPQ	*DRD4*	exon III 48 bp VNTR	n.s.	↓ HA—7R+ vs 7R− carriers
Thome et al. (1999)	94 P (77%), 70 HC (60%)	43.3 ± 10 29.8 ± 8.7	n.a.	DSM-III-R, ICD-10 AD	TPQ	*DRD3*	rs6280	↑ A1 in P vs HC ↓ A2 in P vs HC ↑ A1/A1 in P vs HC	↑ NS in P vs HC with A1/A2 vs A1/A1 P carrying A1/A2: ↑ NS > NS global median of P
Bau et al. (2000)	115 P (100%), 114 HC (50%)	41 (n.a.)	Caucasian	DSM-III-R AD, ASPD	TPQ	*ANKK1*	rs1800497	↑ A1/A1, A1/A2 vs A2/A2 in P vs HC ↑ A1/A2 vs A1/A1, A2/A2 in P vs HC	HA × A1 predicts the number of physiologic dependence symptoms and ASPD symptoms HA—number of antisocial personality symptoms in A1 carriers
Sander et al. (2000)	338 P (82%), 250 HC (39%)	42.9 ± 9.3 44.9 ± 17.2	German	ICD-10 AD (338/338), AD\DPD (56/338)	TPQ	*HTR1B*	rs6296	↑ C/C in AD\DPD vs AD non-DPD	n.s.
Bau et al. (2001)	114 P (n.a.) 112 HC (n.a.)	n.a.	Caucasian	AD (n.a.)	NS (scale n.a.)	*DRD4,* *SLC6A3/DAT1*	exon III 48 bp VNTR, rs28363170 40 bp VNTR	n.s.	↑ NS—DRD4 7/* ^#^ × DAT1 10/10^§^ in AD
Preuss et al. (2001)	135 P (79%)	41.8 ± 8.8	German	DSM-IV, ICD-10 AD (135/135), ASPD (25/135), BPD (23/135)	BIS	*HTR2A*	rs6311	n.s.	↓ BIS—A/A (significant even after excluding ASPD, BDP)
Samochowiec et al. (2002)	72 P (100%)	44 ± 10	German	ICD-10 AD	TPQ	*SLC6A2/NET*	rs5569	n.s.	↑ RD—A
Soyka et al. (2002)	181 P (76%)	40.6 ± 8.8	German	DSM-IV, ICD-10 AD	TCI, SSS, NEO-FFI	*DRD4*	exon III 48 bp VNTR	n.s.	n.s.
Koller et al. (2003)	169 P (100%), 72 HC (100%)	41.8 ± 8 42.2 ± 13.2	German	DSM-IV, ICD-10 AD	BIS, BDHI, LTHA	*MAO-A*	uVNTR 30 bp	n.s.	n.s.
Ponce et al. (2003)	103 P (100%)	41.2 ± 9.72	Spanish	DSM- III-R AD (103/103), IPDE-DSM-IV ASPD (34/103)	/	*ANKK1*	rs1800497	↑ ASPD in A1/A1, A1/A2 vs A2/A2	A1 carriers >risk for ASPD
Soyka et al. (2004a)	164 P (80%)	41.3 ± 9.5	German	DSM-IV, ICD-10 AD (164/164), ASPD (73/164), CD (41/164), ASPD\CD (34/164)	/	*HTR1B*	rs6296	↓ C in CD^#^, ASPD\CD^§^	n.s.
Soyka et al. (2004b)	170 P (75%)	41.3 ± 9.6	German	DSM-IV, ICD-10 AD	TCI	*CRH1*	rs110402, rs171440, rs1396862, rs878886	n.s.	n.s.
Anghelescu et al. (2005)	159 P (68%) 161 HC (60%)	43.4 ± 6.9 41.9 ± 9.1	Caucasian	DSM-IV AD	TCI	*TPH*	rs1800532	↑ A/A, C/C in P vs HC	↓ PE—A/A in HC vs P^§^
Koller et al. (2006)	185 P (81%)	41.55 ± 6.6	German	DSM-IV, ICD-10 AD	NEO-FFI, TCI	*HTR1A*	rs6295	n.s.	n.s.
Lin et al. (2007)	133 P (86%)	38.9 ± 8.9	Han Chinese	DSM-IV ANX\DEP (87/133)	TPQ (NS, HA)	*ANKK1 SLC6A4*	rs1800497 5-HTTLPR	n.s.	↑ NS—DRD2 A1/A1, A1/A2 in ANX\DEP vs PURE ↑ NS—S/S + DRD2 A1/A1 or A1/A2, in ANX\DEP vs PURE ↑ HA—DRD2 A1/A1, A1/A2, A2/A2 in ANX\DEP vs PURE
		40.7 ± 8.4		DSM-IV PURE (46/133)					
	57 HC (n.a.)	n.a.							
Ducci et al. (2008)	168 P (0%), 123 HC (n.a.)	37.8 ± 14.5 (P + HC)	American Indian	DSM-III-R AUD (168/168), AD (86% P), AUD\ASPD (39/168), [CSA (51% P + HC)] G x E	/	*MAO-A*	MAOA-LPR (uVNTR 30 bp), rs1465108, rs909525, rs979605, rs2239448	*Genotypes:* ↑ TC rs1465108, ↑ AG rs909525, ↑ AG rs979605, ↑ AG rs2239448 in AUD vs HC, AUD\ASPD vs HC *Haplotypes:* ↑ 3TGAA in AUD vs HC, AUD\ASPD vs HC	/
						*MAOB*	rs1799836, rs10521432, rs12394221, rs5905512, rs9887047	*Genotypes:* ↑ TC rs10521432, ↑ AG rs12394221, ↑ TC rs9887047 in AUD vs HC *Haplotypes* (no rs1799836): ↑ TGCC in AUD vs HC, AUD\ASPD vs HC	/
Wu et al. (2008)	127 P inmates (100%)	30.7 ± 7.5	Han Chinese	DSM-IV AD\ASPD (43/127), ASPD (84/127)	TPQ (NS, HA)	*ANKK1 SLC6A4*	rs1800497 5-HTTLPR	↑ S/S in AD\ASPD vs ASPD	↑ NS—S/S + DRD2 A1/A1 or A1/A2 in AD\ASPD vs ASPD
Ducci et al. (2009)	284 P inmates (100%), 234 HC (100%)	37.5 0.5 (P + HC)	Finnish	DSM-III-R AUD (284/284), AD (86% AUD), AUD\ASPD (159/284)	/	*HTR3A*	rs1150266, rs2276302, rs3737457, rs117613	/	↓ A rs1150226 in AUD\ASPD vs HC
						*HTR3B*	rs3758987, rs10502180, rs11606194, rs17116121, rs1176744, rs171161138, rs2276307, rs3782025, rs1176761		↑ A rs3782025 in AUD\ASPD vs HC ↑ AA rs3782025 in AUD\ASPD vs HC *Haplotypes:* Block 1 (4 SNPs, w/o rs10502180): n.s. Block 2 (4 SNPs): ↓ GAGT in AUD\ASPD vs HC
Kimura et al. (2009)	460 P (100%)	50.3 ± 8.5	Japanese	DSM-III-R AD	TPQ	*ALDH2*	rs671	n.s.	↑ NS—ALDH2*1/2*2 ↓ HA—ALDH2*1/2*1
Lee et al. (2009)	346 P inmates (100%)	32.6 ± 7.5	Han Chinese	DSM-IV AD\ASPD (132/346), ASPD (162/346)	/	*MAO-A,* *ALDH2*	uVNTR 30 bp, rs671	↑ ALDH2 *1/*2, *2/*2 in ASPD vs AD\ASPD	ALDH2*1/*1 × MAO-A 3R VNTR in AD\ASPD
Anghelescu et al. (2010)	144 P (78%), 144 HC (67%)	44.2 9.7 41.9 10.8	Caucasian	DSM-IV AD (144/144), AD\PCMB (101/144)	NEO-FFI, TCI	*SLC6A3/DAT1*	rs28363170 40 bp VNTR	n.s.	↓ NS—A10/A10 vs A9/A9, A9/A10^#^ in AD vs AD\PCMB, HC ↑ SD—A10/A10 vs A9/A9, A9/A10^#^ in AD vs AD\PCMB, HC
Flory et al. (2011)	151 P (77%)	n.a.	Caucasian	DSM-IV AD	BIS, ZSS-V	*PDYN*	rs35286281 68 bp VNTR L (1,2 repeat; low expression), H (3,4 repeat; high expression)	n.s.	↑ DB—L/L, L/H in AD
Landgren et al. (2011)	84 P (78%), 32 HC (71%)	49 ± 1.1 43 ± 2.1	n.a.	DSM-IV AD, CLON 1	TCI	*GHRL*	rs4684677, rs42451, rs35680, rs34911341, rs696217, rs26802	n.s.	↑ NS—G/G CHRNB3 rs13261190 ^#^ in P ↑ ST—A/A GHRL rs42451^#^ in P
						*GHSR*	rs2948694, rs572169 rs2232165, rs495225		
						*CHRNA3*	rs6495307, rs1317286 rs12443170, rs8042059		
						*CHRNA4*	rs1044396, SNP 12284 rs6011776, rs6010918		
						*CHRNA6*	rs17621710, rs10087172, rs10109429, rs2196129, rs16891604		
						*CHRNB2*	rs2072659, rs2072660		
						*CHRNB3*	rs13261190, rs62518216, rs62518217, rs62518218, rs16891561		
						*ANKK1*	rs1800497		
Lu et al. (2012)	297 P inmates (100%), 244 HC (100%)	36.6 ± 7.4 36 ± 10.2	Han Chinese	DSM-IV AD\ASPD (133/297), ASPD (164/297)	/	*ANKK1, ALDH2*	rs1800497, rs671	↑ ALDH2 *1/*2, *2/*2 in ASPD vs AD + ASPD, HC	DRD2 A1/A1, A1/A2 + ALDH2*1*1 in ASPD vs HC ^#^, ASPD & AD\ASPD vs HC ^#^ DRD2 A1/A1 x ALDH2*1*1 in ASPD, ASPD & AD\ASPD
Wang et al. (2012)	Family-based GWAS 1335 P (50%)	n.a.	Caucasian	DSM-IV AD	TPQ	/	/	/	NS, HA, RD—*ABLIM1* rs727532 (strongest association), *ADK* rs719624, *GPR97* rs727216, *LILRA1* rs272411, *MAO-A* rs979606, *RFX4* rs1882542, *STON2* rs1885604, *TESK* rs1417578, *TIPARP* rs1367311, *THEMIS* rs270015, rs1596762, rs970543, rs1656113, rs1461728, rs265459, rs298881, rs1986644, rs2027148, rs2366517, rs742997 in AD^#^
	Population-based, (replication study), GWAS 1076 P, 1294 HC (43% total sample)	n.a.	Caucasian	DSM-IV AD	TPQ	/	/	/	NS, HA, RD—*ABLIM1* rs727532 in AD *(confirmed)*
Wang et al. (2013)	102 P (89%)	39.1 ± 9	Han Chinese	DSM-IV AD	TPQ (NS, HA)	*SLC6A4* *ANKK1,* *ALDH2*	rs25531, novel allelic variants (XL) [Low functional: SS, SL_G_, L_G_L_G;_ High functional: S/L_A_, L_G_/L_A_, L_A_/L_A_, S/XL, L_A_/XL, L_G_/XL] rs1800497 rs671	↑ S/S, S/L_G_, L_G_/L_G_ in P vs HC	↑ NS—DRD2 A1/A1 or A1/A2 + SS, SL_G_, L_G_L_G_ in P vs HC ↑ NS—ALDH2*1/*2 or *2/*2 + DRD2 A1/A1, A1/A2, A2/A2 in P vs HC ↑ NS—ALDH2*1/*2 or *2/*2 + SS, SL_G_, L_G_L_G_; S/L_A_, L_G_/L_A_, L_A_/L_A_, S/XL, L_A_/XL, L_G_/XL in P vs HC ↑ HA—DRD2 A1/A1 or A1/A2 + SS, SL_G_, L_G_L_G_ in P vs HC ↑ HA—ALDH2*1/*1 + DRD2 A1/A1, A1/A2 in P vs HC ↑ HA—ALDH2*1/*1 + SS, SL_G_, L_G_L_G_ in P vs HC
	111 HC (81%)	36.9 ± 8.7							
Soyka et al. (2013)	293 P (71%)	41.7 ± 8.5	German	DSM-IV, ICD-10 AD (32/293 non-violent crimes; 22/293 violent crimes)	BDHI, LTHA	*COMT*	rs4680	n.s.	n.s.
	190 P (96%)	34.6 ± 8.2	Polish	DSM-IV, ICD-10 AD (24/293 non-violent crimes; 129/293 violent crimes)					
	493 HC (51%)	43.6 ± 16	German						

*AD* alcohol dependence, *AD\ASPD* alcohol dependence with comorbid ASPD, *AD\ DPD* alcohol dependence with comorbid DPD, *ASPD* antisocial personality disorder, *APT* alcohol purchase task, *AUD* alcohol use disorder, *AUDIT* alcohol use disorder identification test, *AUD\ASPD* alcohol use disorder with comorbid ASPD, *BD* binge drinking, *BIS* Barratt Impulsiveness Scale, *BPD* borderline personality disorder, *BDHI* Buss Durkee Hostility Inventory, *CD* conduct disorder, *CSA* childhood sexual abuse, *CHRNA/B* nicotinic acetylcholine receptor α/β gene, *DB* disinhibited behavior, *DPD* dissocial personality disorder, *G* × *E* gene × environment interaction, *GHRL* pro-ghrelin gene, *GHSR* growth hormone secretagogue receptor gene, *IPDE-DSM-IV* International Personality Disorder Examination, *DSM-IV* module, *LTHA* Brown–Goodwin assessment for history of lifetime aggression, *PCMB* psychiatric comorbidities, *PE* persistence, *SD* self-directedness, *SS* sensation seeking, *ST* self-transcendence, *TPH* tryptophan hydroxylase, *ZSS-V* Zuckerman Sensation Seeking Scale

^§^ Non-significant after correction for multiple testing

^#^ Correction for multiple testing

^×^ Interaction

^+^ Stratified genotype analysis

## Results

### Description of studies

A total of 38 studies investigated the relationship between genetics and personality in alcohol use disorder (Tables 1, 2). The majority of studies recruited either inpatients or outpatients diagnosed with alcohol dependence from clinical settings. Thirteen studies examined samples composed only of males. Alcohol dependence was diagnosed according to DSM-III-R, DSM-IV or ICD-10 criteria. Overall, the samples of ADP were commonly diagnosed with comorbid antisocial (ASPD; DSM)/dissocial (DPD; ICD-10) personality disorder. Four studies assessed samples of alcohol-dependent prisoners with comorbid antisocial personality disorder (Wu et al. 2008; Ducci et al. 2009; Lee et al. 2009; Lu et al. 2012). One study assessed ADP with borderline personality disorder (Preuss et al. 2001) and one assessed ADP with conduct disorder (Soyka et al. 2004a, b).

The sample size ranged from 72 to 1335 ADP, and the weighted mean age was 40.5 years. In most of the included studies, participants were Caucasian. Ten studies included Asian ADP (Han Chinese or Japanese), one investigated a sample of American Indians females (Ducci et al. 2008) and one investigated a sample of Afro-American and Hispanic ADP (Herman et al. 2011).

### Personality inventories used in the studies on AUD

Personality traits were commonly evaluated using Cloninger’s Tridimensional Personality Questionnaire (TPQ) (Cloninger et al. 1991) or Temperament and Character Inventory (TCI) (Cloninger et al. 1994). Alternatively, other personality inventories such as the Neuroticism Extraversion Openness Personality Inventory-Five Factor Inventory (NEO-FFI) (Anghelescu et al. 2010; Stoltenberg et al. 2002; Soyka et al. 2002; Koller et al. 2006) and the California Psychological Inventory (CPI-So) (Herman et al. 2011) were used. To measure specific traits, the Barratt Impulsiveness Scale (BIS) (Preuss et al. 2001; Koller et al. 2003; Flory et al. 2011), Buss Durkee Hostility Inventory (BDHI) (Buss and Durkee 1957), Brown-Goodwin assessment for history of lifetime aggression (Balthazart et al. 2004; Soyka et al. 2013; Koller et al. 2003), Sensation Seeking Scales (SSS and ZSS-V) (Zuckerman et al. 1972) (Matsushita et al. 2001; Flory et al. 2011) and California Psychological Inventory (CPI-So) (Herman et al. 2011) were used (Tables 1, 2).

### Polymorphic genetic markers used in the studies on AUD

Ten studies investigated polymorphisms in the *SLC6A4/5HTT* gene (Sander et al. 1998; Stoltenberg et al. 2002; Hallikainen et al. 1999; Wiesbeck et al. 2004; Lin et al. 2007; Herman et al. 2011; Koller et al. 2008; Wu et al. 2008; Wang et al. 2013; Matsushita et al. 2001). Most of these studies focused on the 5-HTTLPR functional polymorphism, whereas few considered other polymorphisms such as rs25531, the 17-bp variable tandem repeat in the second intron (STin2 VNTR) and ‘novel allelic variants’ (XL) (Table 1). Twelve studies focused on dopamine receptors (*DRD2* (and *ANKK1*), *DRD3, DRD4*) (Sander et al. 1997; Bau et al. 1999, 2000, 2001; Thome et al. 1999; Soyka et al. 2002; Ponce et al. 2003; Lin et al. 2007; Wu et al. 2008), six on serotonin receptors (*HTR1A, HTR1B, HTR2A, HTR3A, HTR3B*), four on monoamine oxidase (*MAO-A, MAOB*) and two on the dopamine transporter (*SLC6A3/DAT1*). A smaller number of studies assessed polymorphisms in other candidate genes such as *ALDH2, CRH1, CHRNA, CHRNB2, CHRNB3, COMT, GHRL, GHSR, PDYN, SLC6A2/NET* and *TPH* (Table 2). One study performed a family-based GWAS analysis, which has been replicated in a population-based GWAS analysis (Wang et al. 2012).

### Associations between personality and gene polymorphisms

The trait of NS usually was associated with the short, and less active, variant of the 5-HTTLPR (Table 1). Specifically, carriers of the s allele showed higher NS scores (Sander et al. 1998; Lin et al. 2007; Wu et al. 2008). Additionally, ADP homozygous for the s allele showed higher risk of Cloninger’s Type II alcohol dependence compared to heterozygotes and homozygotes for the l allele, as well as healthy controls (Hallikainen et al. 1999). The s allele was associated with a higher load of sociopathy in female ADP patients, whereas the opposite was observed in men (Herman et al. 2011).

With regard to the dopaminergic system (Table 2), the presence of the seven-repeat allele (7R) of the *DRD4* exon III 48 bp VNTR polymorphism in ADP was found to be associated with lower HA (Bau et al. 1999), and the *DRD4* 7/* genotype interacted with *DAT1* 10/10 genotype and high NS on alcohol consumption (Bau et al. 2001). Moreover, an association between HA scores and ASPD has been shown in *ANKK1* Taq1 A1 ADP carriers (Bau et al. 2000), as well as between ASPD and APD carriers of the A1 allele (Ponce et al. 2003). Additionally, the A1/A1 and A1/A2 genotypes were associated with higher NS in a subgroup of ADP with anxiety and depression (Lin et al. 2007). After stratification by 5-HTTLPR genotype, A1/A1 and A1/A2 genotypes were related to higher NS in ADP with anxiety and depression (Lin et al. 2007), in AD/ASPD (Wu et al. 2008) and in pure ADP (Wang et al. 2013).

Other investigations had observed a modulatory role of *MAO-A* polymorphisms on personality and AUD. The *MAO-A* genotypes have been associated with AD/ASPD (Ducci et al. 2008), and the SNP rs979606 was found to be associated with NS, HA, RD scores in a GWAS (Wang et al. 2012). It should be noted that the sample in the study by Ducci and colleagues is quite specific, including American Indians females who, to a large extent, had been sexually abused during childhood (Ducci et al. 2008). Moreover, the three repeats alleles of the *MAO-A* VNTR polymorphism, in combination with ALDH2*1*1, were more frequent in subjects with AD/ASPD (Lee et al. 2009).

Results regarding other polymorphisms in other candidate genes (e.g., tryptophan hydroxylase *TPH*; serotonin receptors *HTR1A*, *HTR1B*, *HTR2A*, *HTR3A*, *HTR3B*; monoamine-related enzymes and transporters *COMT*, *DRD3*, *MAO-A*, *MAOB*, *SLC6A2*/*NET*; aldehyde dehydrogenase *ALDH2*; prodynorphin *PDYN*; corticotropin-releasing hormone receptor *CRH1*; cholinergic receptors *CHRNB*, *CHRNA*; ghrelin and obestatin prepropeptide *GHRL*; growth hormone secretagogue receptor *GHSR*) were sparse. Furthermore, an association between the SNP rs727532 within the *ABLIM1* gene and NS, HA and RD in ADP was reported in a family-based GWAS and replicated in a population-based sample of ADP (Wang et al. 2012).

## Discussion

The present review scrutinizes the state of the art on the association between genetics and personality in relation to alcohol dependence. We sought to find evidence of genetically driven personality links to define more homogenous subtypes of AUD. Both candidate gene and genome-wide association studies (GWAS), in which the association between genetic variants and standardized measures of personality among alcohol-dependent patients, were considered. However, the results were sparse and no clear conclusions could be drawn.

### Psychobiology of personality

Traits are usually considered to be the fundamental units of an individual’s personality and predispose to a great variety of behaviors. The research area of biological underpinnings of personality traits and personality disorders (PD) has a long history (Charney 1999). Several models have been conceptualized, such as the psychodynamic (Freud 2003), humanistic (Rogers and Dymond 1954), learning (Skinner 1950), cognitive (Bandura 1986) and the trait perspective (Allport 2013).

Of relevance to the present study are the “biological approaches”, among which Eysenck’s (Eysenck and Eysenck 1975), Gray’s (Gray 1991) and Cloninger’s (Cloninger 1987b) are the most studied. Eysenck´s three-factor psychometric model was mainly built on Pavlovian learning principles and identified extraversion, neuroticism and psychoticism as the main traits or personality dimensions (Eysenck and Eysenck 1975). A revised version of Eysenck’s model is Gray´s model, which is based mainly on classical conditioning, but also includes motivational models (motivation and avoidance), impulsivity and anxiety (Gray 1991). These models have been fundamental for the development of Cloninger’s model (Cloninger 1987b), an approach to temperament that merges genetic, neurobiological and neuropharmacological data (Allport 2013; Eysenck and Eysenck 1975). In 1987, Cloninger proposed the existence of three personality systems or temperaments and associated them with specific neural systems: HA (anxious, pessimistic vs outgoing, optimistic) that is correlated with high serotonergic levels; NS (impulsive, quick-tempered vs rigid, slow-tempered) characterized by low dopaminergic activity; and RD (warm, approval seeking vs cold, aloof), similar to extraversion, and correlated with low noradrenergic activity (Cloninger 1986, 1987b). According to the Cloninger’s psychobiological model, personality dimensions are stable traits already present at early age that have high heritability (temperaments) but are also self-regulated during adulthood by the environment (characters) (Cloninger et al. 1993; Josefsson et al. 2013).

Furthermore, adoption studies performed by Cloninger et al. (1981) provided evidence that those personality traits are efficient in identifying two distinct AUD phenotypes. Type I alcoholism, mainly characterized by binge drinking, loss of control and guilt, and low NS, high HA and RD, and a progressive abuse of alcohol. In contrast, type II is characterized by the inability to abstain from alcohol and seems to be more related to criminal behaviors such as fights and arrests, and conduct disorders. These two types also show opposite characteristics with regard to high NS, and low HA and RD (Cloninger et al. 1996).

Additionally, Babor’s studies (Babor et al. 1992) suggested two distinct categories of alcoholics, type A and B. Both Cloninger’s type 1 alcoholism and Babor’s type A are characterized by late age of onset, weaker family history, lower severity of dependence and less psychiatric and social impairment. Likewise, type 2 and type B are more severe, with earlier onset, stronger family history, more impulsivity and antisocial conduct, and comorbid drug abuse. The major difference is Babor’s reliance on self-reported symptoms rather than family history and course of illness in the definition (Babor et al. 1992).

Cloninger’s categorization is still used nowadays (Johnson et al. 2000) and more recent studies attempted proving evidence that those traits are distinguishable in patients with AUD and associated with distinct anatomical and functional brain characteristics.

### Neurotransmitters, brain, personality and AUD

Recently, Belcher and colleagues reviewed the literature on the link between substance use disorders (SUD) and personality, brain regions and neurotransmitters proposing that neuro-temperamental associations, represented as three independent variables, could enhance the vulnerability to SUD (Belcher et al. 2014). According to their hypothesis (Belcher et al., 2014), positive emotionality/extraversion (PEM/E), a trait related to Cloninger’s NS, is modulated by the dopamine system and its neurocircuitries, involving the substantia nigra, ventral tegmental area, striatum and right cingulate gyrus area. On the other hand, negative emotionality/neuroticism (NEM/N), like Cloninger’s HA, is modulated by glutamate and serotonin. It involves the anterior cingulate cortex, ventromedial prefrontal cortex, amygdala and insula, whereas constraint (CON), modulated by glutamate and dopamine, involves the pre-supplementary motor area, right inferior frontal gyrus, striatum and subthalamic nucleus.

Despite that Cloninger’s and Belcher’s frameworks share some similarities, the involvement of dopamine in NS or PEM/E and of serotonin in HA or NEM/N, they also show some differences. Cloninger expected high levels of dopamine and serotonin in type I (high HA, high RD, low NS) and low levels of serotonin activity in type II (high NS, low HA, low RD) (Cloninger 1987a). In fact, Cloninger´s type II (high NS, low HA, low RD) would represent a high vulnerability phenotype, while according to Belcher and colleagues it would constitute a resilience factor for SUD (Belcher et al. 2014). This discrepancy can be reconciled by observing that extreme high or extreme low HA increases the risk of type II alcoholism; thus, type II can be considered as a mixture of antisocial individuals, some of whom are low (antisocial or adventurous types) and some high in HA (i.e., borderline or explosive types) (Cloninger et al. 1995, 1988). Alternatively, since Belcher and colleagues find evidences generally on SUD, it could be speculated that their hypothesis may not be specifically valid for alcohol. Furthermore, both type I and type II represent a vulnerability factor model even though type I development of addiction is described as progressive and less dramatic compared to type II that seems to lead to a fast shift to addiction (Cloninger et al. 1995).

To date, 38 studies have investigated personality traits, mainly assessed using the Cloninger’s TPQ and TCI, in relation with polymorphisms of candidate genes of neurotransmitters systems in alcohol-dependent patients. As summarized in the tables, the analysis on the relation between genotype and personality traits in AUD has highlighted contradictory results, regarding traits, genes and personality.

### Serotonin, personality and AUD

Particular attention was given to the serotonin transporter gene-linked polymorphic region (5-HTTLPR) (Heils et al. 1996). The 5HT gene regulates the expression of the serotonin transporter (named 5-HTT or SERT or SLC6A4) that modulates the termination of the serotonin signal at the synaptic level through a reuptake mechanism (Amara and Kuhar 1993). The 5-HTTLPR consists of an insertion/deletion of a repeat motif, with each repeat being 22–23 base pair long, occurring in the promoter region of the *5HTT* gene. Usually, the two common alleles are referred to as long (l) and short (s), and consist of 14- and 16-repeat alleles, respectively (Nakamura et al. 2000). The functional difference between l and s is a two-/threefold higher transcriptional activity associated with the l allele, as assessed in lymphoblast cell lines (Heils et al. 1996; Hahn and Blakely 2007; Lesch and Gutknecht 2005). It has been shown that individuals with the l allele have higher levels of 5-HTT in platelets and in the brain (Hu et al. 2006). Moreover, one rare variation of this polymorphism has been proved to be functional: an adenine to guanine (A/G) single nucleotide polymorphism (SNP), rs25531, within the l allele (Nakamura et al. 2000; Hu et al. 2006; Hahn and Blakely 2007). This SNP was found to modify the transcriptional activity of *5*-*HTT,* with the l_G_ allele having equivalent activity to the s allele, and heterozygotes, l_A_/l_G_, s/l and s/l_G_, having intermediate activity compared to s/s and l_A_/l_A_ (Hu et al. 2006).

Carrying the s allele has been associated not only with alcohol dependence (Rubens et al. 2016; Feinn et al. 2005), but also with stress reactivity, impulsivity, anxiety and mood disorders (Reif and Lesch 2003; Walderhaug et al. 2007; Calati et al. 2011; Munafò et al. 2008). The findings of the studies included in the present review cannot support any conclusion regarding the association between 5-HTTLPR and personality traits in relation to alcohol dependence (Table 1). A trend of association was found between NS and homozygosity for the s allele in alcohol-dependent individuals with dissocial personality disorder (Sander et al. 1998), anxiety, depression (Lin et al. 2007) and antisocial personality disorder (Wu et al. 2008). Inversely, a borderline significant association between high levels of HA and heterozygosity has been shown in alcohol-dependent patients with anxiety and depression (Koller et al. 2008; Lin et al. 2007; Wiesbeck et al. 2004). Only one study has found a correlation between homozygosity for the s allele and Cloninger´s alcoholism type II (Hallikainen et al. 1999). Nevertheless, complementary evidence indeed points to the role of serotonin in the development of AUD.

Personality traits and vulnerability gene variants have a variety of ways to compromise mental health, as etiopathogenetic pathways comprise multiple steps. A pharmacologist would emphasize that AUD quite obviously develops as a consequence of early onset of alcohol use (Liang and Chikritzhs 2015). It is self-evident that personality traits, as well as several of the gene variants, may also exert their effect in the development of AUD via promoting early excessive alcohol use. The 5-HTTLPR genotype has been associated with alcohol use in many studies (Rubens et al. 2016) and, given the evidence of the association between the s allele, neuroticism, higher sensitivity to stress and high reactivity of corpus amygdala in response to aversive stimuli, it is tempting to speculate that the association between the s allele and AUD, if present in a given population, may reflect this aspect of development of the disorder. According to this line of thought, some of the negative findings may well be explained by the existence of highly adaptive behavioral mechanisms that in turn help to understand the persistence of the common “vulnerability variants”, otherwise seen as “flexibility variants” (Belsky et al. 2009) in the gene pool. The variable and changeable environmental demands are likely to cause the rather weak, while statistically significant associations between personality and gene variants with alcohol use and the development of AUD. An explanation for that is suggested in a study on two birth cohorts that represent the general population but were collected at different times with several years in between. In both cohorts, alcohol use was more frequent in s allele carriers of 5-HTTLPR (Merenäkk et al. 2011; Vaht et al. 2014), but this genotype effect appeared at later age in one of the cohorts. Furthermore, a highly significant genotype × birth cohort interaction effect was found on the age of onset of use of alcohol. While the s/s-homozygote girls were the last group to begin with alcohol in one cohort, they were the earliest, even ahead of boys, in the other birth cohort that grew up when alcohol had become much easier to obtain and more widely used (Vaht et al. 2014). High neuroticism and social desirability, both associated with the s allele of the 5-HTTLPR, are likely to either suppress or facilitate alcohol use as the societal norms radically change and hence to have the potential to produce opposite effects under distinct environmental conditions even within family or community settings.

Through modulation of reward behavioral inhibition and affect, the serotonergic system exerts an important role in addiction (Goodman 2008; Kranz et al. 2010), mainly indirectly through its connections with the dopamine system. Attention has been given to the role of 5-HT1B, 5-HT2A and 5-HT2C receptors in seeking behavior, alcohol consumption and preference in studies of rodents (Pentkowski et al. 2010; Pockros et al. 2011; Czachowski 2005; Furay et al. 2011; Goodman 2008; Rezvani et al. 2014) as well as in humans (Reif and Lesch 2003). In the present review, some studies have investigated the role of *HTR1B* and *HTR1A* genes, however, without significant results (Sander et al. 2000; Preuss et al. 2001; Koller et al. 2006). On the other hand, studies have shown an association between A/A *HTR2A* genotype and low levels of behavioral inhibition system trait (Preuss et al. 2001) and between A/A *HT3B* genotype in alcohol-dependent patients with ASPD (Ducci et al. 2009). Moreover, it has been demonstrated that 5-HT-mediated reduced availability of dopamine enhances seeking behaviors and addiction, in line with low levels of HA and low levels of 5HT, but high levels of NS and low levels of dopamine being associated with Cloninger´s type II. In the present review, two studies on the *DRD2* gene reported a relation between high levels of NS in alcohol-dependent patients carrying the A1 allele, after stratification by 5-HTTLPR genotype (Wu et al. 2008; Wang et al. 2013).

Addiction also shares features with impulse control disorders, characterized by feelings of tension before performing the act, and pleasure and relief once the performance is over (Koob 2011). Personality models depict impulsivity as characterized by various traits such as NS, venturesomeness and sensation seeking, which have been associated with AUD (Stevens et al. 2014). Additionally, a bidirectional interaction between addiction and impulsivity has been reported, with not only increased levels of impulsivity leading to acquire alcohol abuse behavioral patterns, such as seeking and dysregulated intake, but also abuse enhancing impulsivity (Perry and Carroll 2008). High levels of impulsivity have been found in AUD patients (Rogers et al. 2010; Whiteside and Lynam 2003). In line with the hypothesis that serotonin inhibitory control on impulsive behavior is involved in alcohol abuse (Collins and Schlenger 1988), impulsive individuals display lower cerebrospinal fluid levels of the serotonin metabolite 5-HIAA (Goodman 2008; Linnoila et al. 1983).

Serotonin is also involved in the stress system, and compromised stress regulation involves amygdala-mediated effects on the 5-HT pathway projecting from the raphe nuclei to the paraventricular nucleus (Weidenfeld et al. 2002). Though contradictory findings exist, the 5-HTTLPR s allele has been shown to modulate stress sensitivity, amygdala functioning and its functional connectivity with ACC (Hariri et al. 2002; Canli and Lesch 2007). Increased amygdala activity implies enhanced aversive conditioning to context, and its persistent activation leads to increased negative emotional memories (Pezawas et al. 2005), all well-known processes implied in addiction (Koob 2011). Indeed, enhanced amygdala activity has been proposed as a predictor of drinking behavior (Greenberg et al. 1999) and has been linked with behaviors that are hallmarks of neuroticism, such as fear, worry, depression, stress and anxiety, and that are typically found in alcohol addiction and influence relapse (Koob 2011). Reduced fronto-amygdalar connectivity has been shown in the presence of neuroticism in healthy subjects (Kennis et al. 2013) and AUD patients (Belcher et al. 2014). It is thus plausible that high levels of serotonin and enhanced amygdala activity are the biological substrate of HA that represents a susceptibility endophenotype of AUD (Belcher et al. 2014). However, the literature on alcohol as an outcome of the interaction between 5-HTTLPR and stress is discordant both in human and non-human primates (reviewed by Todkar et al. (2013).

### Dopamine, noradrenaline, personality and AUD

The dopaminergic system exerts a major role in addiction, regulating reward prediction, incentive salience and motivated behavior, as well as impulsivity and mood (Koob 2011). Indeed, postsynaptic D1 and D5 receptors have a role in motivation processes, and presynaptic receptors D2, D3, D4 in behavioral inhibition (Goodman 2008). Particular attention has been given to the TaqI polymorphism on the *ANKK1* gene. Patients carrying the A1 allele show low striatal levels of D2 receptors, associated with seeking for reinforcers and alcohol consumption (Comings and Blum 2000). In addition, association have been found between *DRD2* and *DRD4* polymorphisms and novelty seeking, substance abuse and impulsivity behavior (Belcher et al. 2014; Ebstein et al. 1996; Noble et al. 1998; Lee et al. 2003a), as well as schizophrenia and mood disorders (Blum et al. 1996). Nevertheless, in the present review, studies of polymorphisms on the *DRD2* and *DRD4* genes yield inconsistent results. Furthermore, noradrenaline regulates the stress system release and increases during withdrawal. Among the reviewed studies, one study on the noradrenaline transporter gene (*NET*) found an association between the rs5569 polymorphism of the gene and reward dependence personality trait in alcoholics (Samochowiec et al. 2002). Future studies should also investigate polymorphisms of systems such as the GABAergic and glutamatergic, which play an important role in AUD.

### Confounding factors

Several covariates such as comorbidity, gene–environment interactions, sex and ethnicity need to be considered. As summarized in the tables, comorbidity between AUD and depression and anxiety disorders is common, and shared genetic vulnerability is plausible (Cross-Disorder Group of the Psychiatric Genomics 2013). Despite the discordance and sparse nature of findings of the herein reviewed studies, it is obvious that a great number of genes should be involved in AUD, with likely different genes being of greater importance at various stages of addiction (Koob and Volkow 2010), and that genetic and neural underpinnings of personality traits contribute not only to AUD but also to other psychiatric phenotypic conditions. In fact, a recent genome-wide meta-analysis has shown a relation between genetic loci, personality traits and psychiatric conditions (Lo et al. 2016). Furthermore, overlapping genetic constructs across psychiatric disorders are beginning to be demonstrated (Lee et al. 2013; Smoller et al. 2013). Additionally, psychiatric conditions, as presently defined, have been associated with both the higher or lower end of a personality trait, thus making the neurobiology dissection of AUD and comorbid disorders difficult. For instance, low *MAO*-*A* expression genotype has been linked to AUD-related behaviors such as high impulsivity, aggression and violence, while the high activity MAO-A genotype has been associated with anxiety and depression (Naoi et al. 2016). Thus, both gene variants seem to constitute vulnerability factors, although the underlying developmental trajectories would probably reflect two different subtypes of AUD.

It must also be taken into account that various personality-linked types of AUD are influenced by genes and environment interacting with each other and that those two factors influence each other in a way that is yet difficult to understand. Epigenetics is likely to be the molecular missing link behind this interaction (Shukla et al. 2008). Epigenetics refers to processes that influence the regulation of DNA transcription without changing the original sequence itself. Epigenetic marks (e.g., DNA methylation), which are experience dependent (e.g., early life stress) as well as influenced by genetic architecture, can leave long-lasting changes at the neurobiological and behavioral levels. Accordingly, Philibert and colleagues found that MAO-A gene methylation is not associated with antisocial personality disorder but with AUD in women, and in a *MAO-A* genotype-dependent manner (Philibert et al. 2008). However, genetic associations with personality profiles and AUD should also consider differential susceptibility of specific candidate genes operating in both adverse and beneficial contexts, which results in extraordinarily poor or remarkably positive phenotypes in certain subpopulations as a consequence of psychosocial and physical environmental conditions (Boyce 2016). Recently, it has been shown that individuals carrying combinations of susceptible variants of the *BDNF*, *5*-*HTT* and MAO-A genes are less delinquent when controlling for the interaction of positive parent–child relationship (Nilsson et al. 2014). On the other hand, functionally opposite genotypic combinations are associated with delinquent behavior in the presence of maltreatment and sexual abuse, and the experience of a positive environment modulates the experience of a bad environment in an E × E interaction manner on top of the G × E interaction (Nilsson et al. 2014). Furthermore, it can be suggested that genetic associations of clinical case–control studies mimic G × E studies, since the patients most often have been more exposed to negative life events associated with the phenotype of interest. Therefore, clinical subpopulations displaying AUD with an antisocial component could be very much different in their genetic and environmental makeup than another subpopulation with AUD and anxiety traits, and respond differently to treatment (Karno and Longabaugh 2004). This implies that the individual characteristics may better fit with some environments than with others, and hereditary capacities may only become manifest in challenging or responsive environments (Reiss et al. 2013).

Concerning personality, although character traits are as heritable as temperament (Gillespie et al. 2003), character traits have been shown to be more influenced by sociocultural influences than temperament (Cloninger et al. 1993; Cloninger 1987b; Josefsson et al. 2013). A recent study on adolescents has suggested that personality traits may play a role in driving the individual to the initial use of alcohol as well as choosing between different environments (Heinrich et al. 2016). Early environmental experiences, like traumatic events during childhood, have been associated with high vulnerability to AUD (De Bellis 2002). Recently, a multidisciplinary study systematically examined several components of alcohol misuse in adolescents, such as environmental factors, personality, candidate genes, brain structure and functionality. It was reported that NS was strongly associated with personal life events and binge drinking behaviors, while consciousness was lower in current and future binge adolescents (Whelan et al. 2014). A more recent longitudinal study showed that the adolescents homozygous for the A allele of the vesicular monoamine transporter (*VMAT1*) showed low levels of neuroticism, anxiety and impulsivity, but also were less likely to be diagnosed with alcohol dependence in the following 5 years, suggesting that the gene has a role in resiliency to negative emotions (Vaht et al. 2016). Coping, a common reason for drinking (Comasco et al. 2010), has indeed been found to mediate the relation between AUD and certain personality traits, thus playing a critical role in vulnerability to AUD (Cooper et al. 2000; Kuntsche et al. 2006; Tragesser et al. 2007). Furthermore, life events, such as physical or sexual abuse in adolescence, stress and opportunity to drink, can facilitate the expression of a silent genetic susceptibility (Nestler 2000). Serotonin is a neurotransmitter involved in brain development, from neurogenesis to neuronal differentiation in early life, and in the maintenance and plasticity of the brain in adulthood (Cases et al. 1996; Gaspar et al. 2003; Nordquist and Oreland 2010); thus, prenatal exposure and early life events such as traumatic experiences or early approaches to alcohol may interfere with those processes.

Regarding sex differences, recent evidences show that sex plays a role in alcohol consumption and addiction (Sanchis‐Segura and Becker 2016). In fact, the heritability of AUD is higher in males than in females (Khemiri et al. 2016; Prescott 2002) and men display higher alcohol consumption and more than a double prevalence of lifetime alcohol dependence compared to women, who on the other hand are more likely to display more negative physical and mental health outcomes due to AUD than men (Nolen-Hoeksema 2004). Furthermore, Cloninger’s alcoholic subtypes have shown different personality trait differences related to sex, with type II primarily found in males (Knorring and Oreland 1996). Additionally, while women score higher on RD and HA than men (Miettunen et al. 2007), men score higher on sensation seeking (Cross et al. 2013), and while women have higher scores on neuroticism, men tend to have higher scores on psychoticism (Weijers et al. 2003). Accordingly, a recent study has found high levels of neuroticism and anxiety in women and high levels of NS to be related to binge drinking behaviors in university students (Adan et al. 2016). Those evidences highlight the fact that traits may predispose to AUD in a sex-dependent manner. Further investigations should be performed to study brain development and hormonal variations that could play a role at different ages (Toffoletto et al. 2014; Sanchis‐Segura and Becker 2016). In the present review, as also pointed out by other authors (Nolen-Hoeksema 2004), most studies neglected possible sex effects by assessing all-male samples, and, in case that both sexes were considered, the female samples were much smaller. In addition, it has recently been proposed that the 5-HTTLPR gene may influence 5-HTT expression according to sex, due to the evidence that the gene affects 5-HT functioning in different ways among men and women in the development of depression or affective dysregulation (Gressier et al. 2016; Sjöberg et al. 2006; Brummett et al. 2008). In fact, while cultural-driven gender differences can change, biological sex differences remain, but are often neglected.

Lastly, another explanation about the inconsistencies of the data could be due to ethnicity-based differences in genotype frequencies (Pascale 2015). With regard to ethnicity-based differences in the frequency of alleles of the 5-HTTLPR (Hahn and Blakely 2007; Edenberg et al. 1998; Gelernter et al. 1997), the l allele is less frequent in Asian compared to European-American populations (Lee et al. 2003b; Nakamura et al. 2000), while the s allele frequency has been found to be low in African Americans (0.25), intermediate in Caucasians of American and Finnish origin (0.35–0.40) and high in American Indians (0.64–0.66). Furthermore, the l_G_ was almost absent in two populations of American Indians (Hu et al. 2006).

### Future perspectives and conclusions

AUD is a biopsychosocial phenomenon influenced by several variables, e.g., genes, personality, environmental events, brain structure, emotion and cognitive functioning, socio-demographic factors, age and sex. In fact, while there is a large agreement that the genetic makeup plays an important role on the risk of AUD, an assumption mainly based on twin and family studies (Goldman et al. 2005), neither candidate gene approaches nor GWAS have substantially advanced knowledge. On the other hand, studies investigating the genetic contribution to a phenotype, by comparing shared and non-shared environment, did not consider gene–environment interactions (Munafo et al. 2014). In addition, the complexity of the relations between genes and personality is worsened by non-linear dynamic relations. In fact, geneticists have introduced the concepts of “multifinality” and “equifinality” to describe that either the same or different genetic networks may lead to, respectively, different or similar behaviors involved in complex disorders (Arnedo et al. 2015).

Future studies should therefore control for well-known confounding variables such as sex, parental and personal history of the participants, socio-demographic factors, stressful life events and comorbid symptoms. Ideally, validated methodologies and structured interviews aiming at in-depth phenotyping could help to define the grade in which behavior and biology are connected, most likely closer to the research domain criteria than to nosological constructs such as the DSM criteria and categories. Despite practical limitations, longitudinal studies comprising larger but also more homogenous samples would additionally allow performing within-subject statistics that could help clarify dynamic trajectories toward AUD and whole-genome-based analyses. Only one GWAS study has been performed to date; the strongest association reported was between the Actin Binding LIM Protein 1 (*ABLIM1*) gene and NS, HA and RD in AUD patients (Wang et al. 2012). However, the role of this gene in AUD remains to be explored. Finally, it is equally important to investigate resilient personality dimensions, their relation with reasons for not drinking, and genetic and environmental correlates (Feder et al. 2009).

In conclusion, evidence from family and twin studies supports a genetic basis for AUD, but the clinical heterogeneity among AUD patients has made research on risk and resilience factors difficult. This can be easily understood when considering that AUD can be a consequence of an impulsive, sensation-seeking and extroverted personality, as well as of an anxiety-prone and introverted personality, which by several neurobiological markers might represent two different extremes with regard to personality. This complexity of AUD is reflected in many efforts through the years to construct psycho-biological models to classify clinically relevant AUD subtypes to improve the clinical management. Personality is strongly regulated by genetic factors and should therefore be a top candidate intermediate correlate for the dissection of the genetic underpinnings of different subtypes of AUD (Fig. 1).
